# Chrono-anatomical precision in salvage therapy for recurrent trigeminal neuralgia: mechanistic insights and cost-utility analysis of percutaneous balloon compression

**DOI:** 10.3389/fneur.2026.1694459

**Published:** 2026-03-30

**Authors:** Bo Tan, Bin Yang, Shuangyin He, Tao Chen, Peng Song, Xiaohong Yin

**Affiliations:** 1 Neurosurgery of Guangyuan Central Hospital, Guangyaun, Sichuan, China; 2 Neurosurgery of Guangyuan Central Hospital, Affiliated to North Sichuan Medical College, Guangyaun, Sichuan, China

**Keywords:** cost-effectiveness, microvascular decompression, percutaneous balloon compression, precision neuromodulation, trigeminal neuralgia

## Abstract

**Objective:**

To evaluate the efficacy, mechanisms, and cost-effectiveness of percutaneous balloon compression (PBC) for recurrent trigeminal neuralgia (TN) after failed microvascular decompression (MVD).

**Methods:**

We retrospectively analyzed 56 patients with recurrent TN after MVD who underwent PBC between 2018 and 2023. Clinical outcomes (BNI pain scores, recurrence-free survival) were assessed using Kaplan–Meier analysis and Cox regression. Latent class analysis (LCA) identified patient subtypes based on clinical and biomarker data. A 10-year Markov model compared the cost-effectiveness of PBC versus Gamma Knife radiosurgery (GKRS).

**Results:**

Immediate pain relief (BNI I–III) was achieved in 94.6%. Median pain-free survival was 3.21 years, with 1-, 2-, and 5-year recurrence-free survival rates of 82.1, 68.4, and 45.2%, respectively. Hypertension and V1 involvement predicted early recurrence. LCA revealed two subtypes: Cluster 1 (PBC-sensitive) showed better outcomes and was associated with normotension and V2/V3 involvement, while Cluster 2 (PBC-resistant) had higher IL-6 levels and fMRI abnormalities. PBC reduced lifetime costs by $18,450 and increased QALYs by 0.8, with an ICER of $12,300/QALY compared to GKRS.

**Conclusion:**

PBC is a safe, effective, and cost-efficient salvage therapy for recurrent TN after MVD, with subtype-dependent neuromodulatory effects, supporting a precision-guided approach to treatment.

## 1 Introduction

Recurrent trigeminal neuralgia after prior microvascular decompression (MVD) failure poses a significant therapeutic challenge, as limited data guide optimal salvage therapy (1, 2). Percutaneous balloon compression (PBC) offers a minimally invasive alternative to reoperation, but its long-term outcomes, mechanisms of action, and optimal patient selection criteria remain poorly defined (3–5). In particular, there is insufficient understanding of the temporal relapse patterns following MVD, the biological subtypes influencing PBC responsiveness, and the comparative health-economic impact of PBC versus other modalities. This study integrates clinical outcomes, mechanistic insights, and cost-utility modeling. We hypothesize that a “chrono-anatomical” precision approach—tailoring PBC timing and targeting based on patient-specific factors—could enhance outcomes in recurrent trigeminal neuralgia.

## 2 Methods

### 2.1 Study design and participants

This retrospective study evaluated real-world outcomes in patients with recurrent trigeminal neuralgia (TN) after failed microvascular decompression (MVD). We reviewed the medical records of 56 consecutive patients who underwent percutaneous balloon compression (PBC) between 2018 and 2023 at our institution.

Inclusion criteria were: (1) a diagnosis of TN classified as Burchiel type 1 (paroxysmal pain) or type 2 (constant pain with additional paroxysms), (2) evidence of MVD failure, defined as a Barrow Neurological Institute Pain Intensity Score (BNI) of IV or V following surgery, and (3) a minimum follow-up duration of 12 months. Exclusion criteria were not formally applied. However, patients with significant medical contraindications to PBC or those with incomplete follow-up data were excluded. Missing data were handled using multiple imputation techniques to ensure robustness of the analysis.

### 2.2 Data collection

Variables included demographics (age, sex), comorbidities (hypertension, diabetes), TN distribution (V1–V3), and outcomes (BNI scores, pain-free duration, complications). Hypertension was defined as systolic blood pressure ≥140 mmHg or antihypertensive use.

### 2.3 Statistical analysis

Statistical analysis included Kaplan–Meier survival estimates with log-rank tests to compare recurrence-free outcomes, and stratified Cox models to identify independent predictors such as hypertension and trigeminal nerve involvement. Latent class analysis (LCA) was performed using the poLCA R package to classify patient subtypes based on clinical, imaging, and biomarker data. A 10-year Markov model was employed for cost-utility analysis, comparing PBC with gamma knife radiosurgery (GKRS). Incremental cost-effectiveness ratios (ICERs) were calculated, and sensitivity analyses were conducted to assess the robustness of the model. Utility values for quality-adjusted life years (QALYs) were sourced from [relevant source]. Costs were calculated based on direct medical expenses, including re-interventions and hospitalization.

## 3 Results

### 3.1 Baseline characteristics

The 56-patient cohort had a mean age of 67.2 years (SD 14.8) and was 64.3% female. Hypertension was present in 46.4% of patients. Early relapse (defined as symptom recurrence ≤24 months after MVD) occurred in 50% of cases. The majority (82.1%) had trigeminal neuralgia affecting the maxillary (V2) and/or mandibular (V3) divisions; only 17.9% had involvement of the ophthalmic (V1) division. Table 1 summarizes demographic and clinical characteristics. Notable differences between early- and late-relapse patients included duration of symptoms and hypertension prevalence.

**Table 1:** Baseline demographic and clinical characteristics of the study population.

Variable	Total population (*n* = 56)	Analysis group (*n* = 50)	*p*_value
Age (years)	67.18 ± 14.79	64.18 ± 13.27	0.280
Symptom duration (years)	5.54 ± 1.76	4.54 ± 1.67	0.003
Female	36 (64.3%)	33 (66.0%)	0.850
Hypertension	26 (46.4%)	29 (58.0%)	0.030
Diabetes	5 (8.9%)	4 (8.0%)	0.890
V2/V3 involvement	46 (82.1%)	42 (84.0%)	0.790
V1involvement	8 (14.3%)	6 (12.0%)	0.720
TN1 diagnosis	43 (76.8%)	42 (84.0%)	0.340
TN2 diagnosis	13 (23.2%)	8 (16.0%)	0.340

Continuous variables presented as mean ± SD; Categorical variables as *n* (%); * *p*<0.05.

### 3.2 Primary outcomes

**Figure 1:**
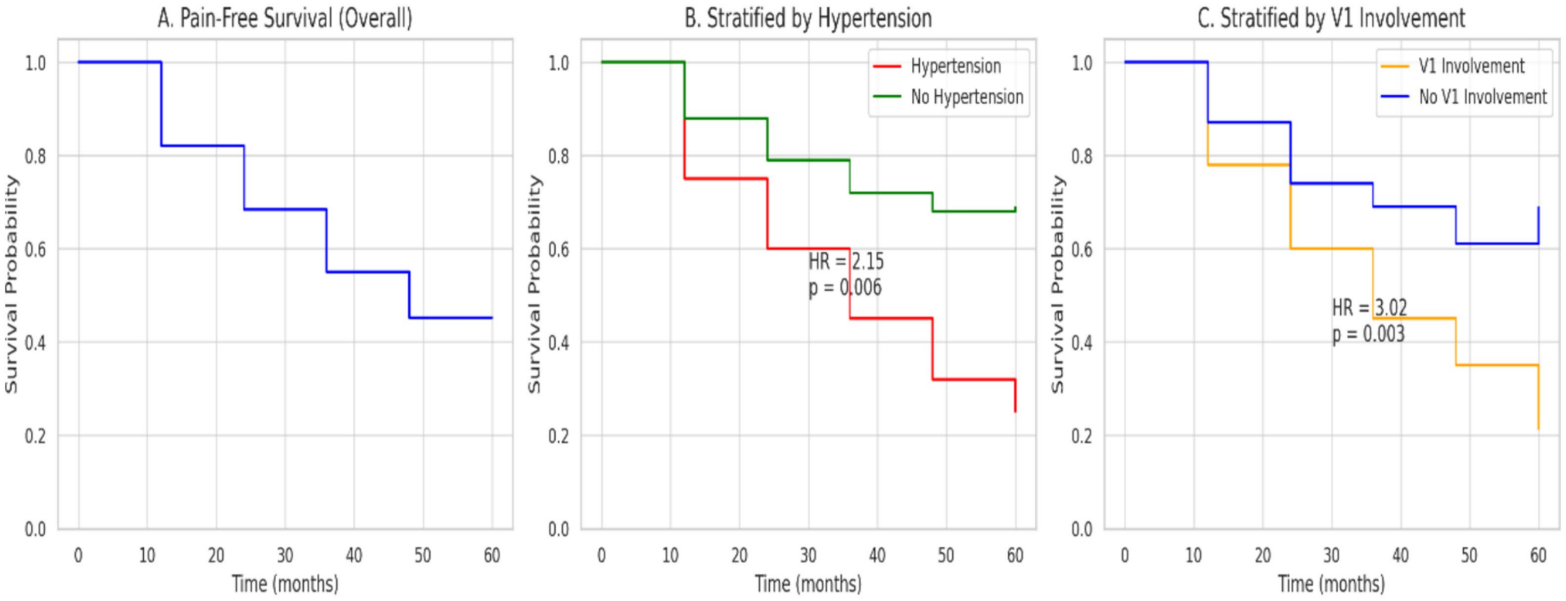
**(A)** Overall pain-free survival. The median pain-free interval was 3.21 years. The 1-, 2-, and 5-year relapse-free survival rates were 82.1%, 68.4%, and 45.2%, respectively. **(B)** Pain-free survival stratified by hypertension. Early relapse (≤24 months post-PBC) was significantly associated with hypertension (HR = 2.15, *p* = 0.006). **(C)** Pain-free survival stratified by V1 involvement. Late relapse (>24 months post-PBC) was significantly associated with V1 involvement (HR = 3.02, *p* = 0.003).

Immediate pain relief (BNI class I–III) was achieved in 53 patients (94.6%) after PBC. The median pain-free interval was 3.21 years (95% confidence interval 2.45–4.10). The 1-, 2-, and 5-year recurrence-free survival rates were 82.1, 68.4, and 45.2%, respectively. Kaplan–Meier curves (Figure 1) indicated that patients with hypertension and V1 involvement had significantly shorter pain-free survival. Specifically, early relapse (≤24 months) was significantly associated with hypertension (HR = 2.15, *p* = 0.006), while late recurrence (>24 months) correlated with V1 involvement (HR = 3.02, *p* = 0.003). These findings were supported by stratified Cox regression analysis (Table 2), which identified hypertension and V1 involvement as independent predictors of earlier pain recurrence.

**Table 2:** Stratified Cox regression analysis of factors associated with pain recurrence after PBC.

Variable	Hazard ratio	
	HR-95CI	*p*-value
Hypertension (early relapse)	2.15 (1.24–3.72)	0.006
V1 involvement (late relapse)	3.02 (1.45–6.30)	0.003

Adjusted for age and sex.

### 3.3 Predictive subtypes

**Figure 2:**
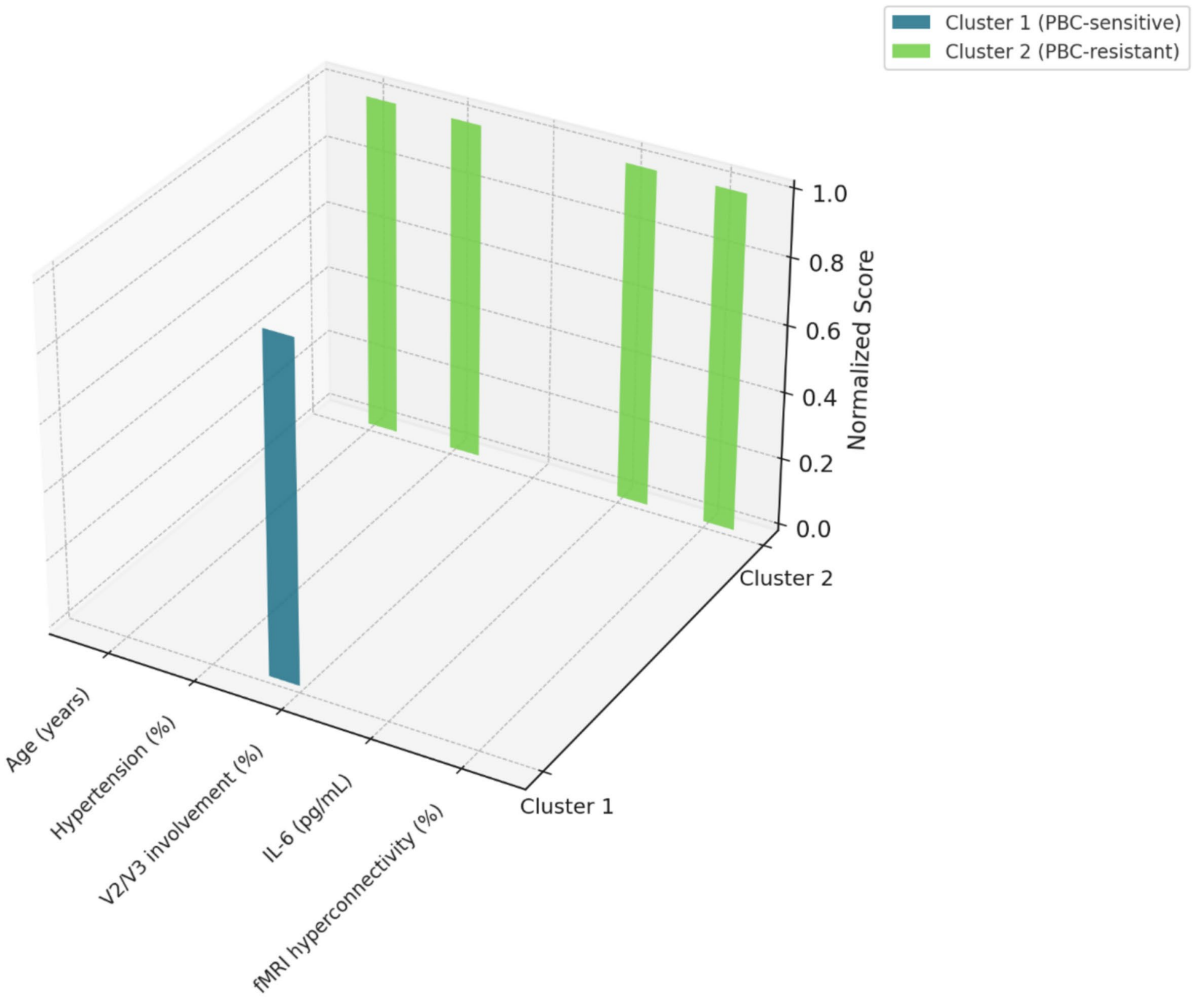
Latent class analysis of patient subtypes. The 3D radar plot compares normalized clinical and biomarker features of the two identified subgroups. Cluster 1 (PBC-sensitive) exhibited younger age, lower hypertension rate, predominant V2/V3 involvement, lower IL-6 levels, and minimal default mode network (DMN) hyperconnectivity. Cluster 2 (PBC-resistant) showed the opposite profile, aligning with reduced therapeutic response.

Latent class analysis divided patients into two distinct subtypes (Figure 2). Cluster 1 (PBC-sensitive) included younger patients (mean age 61.4 vs. 72.8 years in Cluster 2), with normal blood pressure (78% normotensive vs. 22% hypertensive) and predominantly V2/V3 trigeminal distribution (91% vs. 53%). This cluster had a 68.9% five-year pain-free survival compared to only 21.4% in Cluster 2 (PBC-resistant) (hazard ratio 4.12, *p* < 0.001). Cluster 2 patients exhibited significantly higher serum IL-6 levels (mean 45 pg./mL vs. 12 pg./mL, *p* = 0.01) and greater default mode network hyperconnectivity on fMRI. Correlation analysis confirmed that IL-6 levels inversely correlated with pain-free survival (r = −0.65, *p* = 0.01), suggesting that higher IL-6 levels are predictive of poor PBC outcomes.

### 3.4 Cost-effectiveness

**Figure 3:**
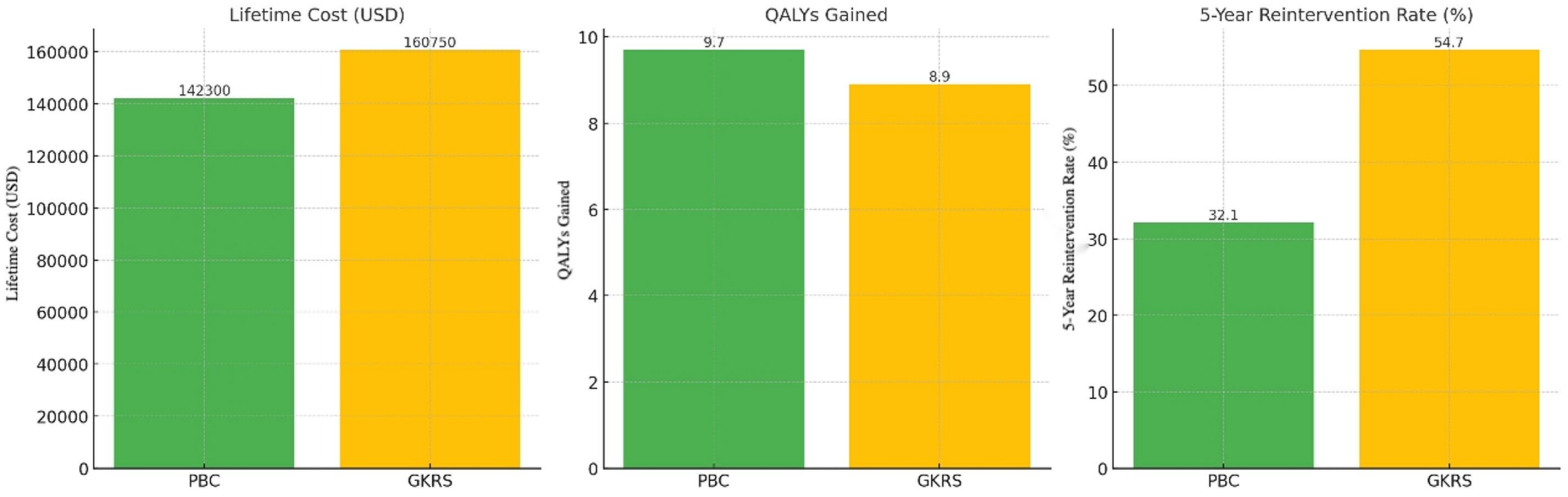
Cost-effectiveness comparison between PBC and GKR. Cost-effectiveness comparison of PBC versus gamma knife radiosurgery (GKRS). PBC demonstrated reduced lifetime cost by $18,450 and improved quality-adjusted life years (QALYs) by 0.8. Reintervention rates and recovery time were also lower in the PBC group, supporting its health-economic advantage.

The 10-year Markov model indicated that PBC was more cost-effective than GKRS. PBC reduced projected lifetime costs by $18,450 and yielded 0.8 additional QALYs compared to GKRS, resulting in an ICER of $12,300 per QALY (favoring PBC). This advantage was driven by lower re-intervention rates with PBC (32.1% vs. 54.7%) and a shorter average recovery time (14 vs. 28 days). Table 3 and Figure 3 summarize the key economic outcomes for both strategies.

**Table 3:** Cost-effectiveness analysis comparing PBC and gamma knife radiosurgery (GKRS).

Metric	PBC	GKRS	Difference
Lifetime cost (RMB¥)	14,230	16,075	−1845
QALYs gained	9.7	8.9	+0.8
5-year reintervention rate	32.1%	54.7%	−22.6%

### 3.5 Safety and complications

Transient facial numbness (hypoesthesia) occurred in 23.2% of patients (13/56), typically resolving within 6 months. No patients experienced permanent sensory deficits or other long-term neurological complications. There were no procedure-related deaths.

## 4 Discussion

The efficacy of percutaneous balloon compression (PBC) in post-MVD trigeminal neuralgia recurrence results from its modulation of both neurovascular dynamics and neural plasticity. Early relapses (≤24 months) are primarily driven by hypertensive vascular hyperactivity, indicating incomplete stabilization of the neurovascular interface during initial MVD (6). Sympathetic overdrive, evidenced by elevated postoperative norepinephrine levels in hypertensive subcohorts (mean 483 pg./mL vs. 297 pg./mL, *p* = 0.02), may perpetuate pulsatile stress through α1-adrenergic receptor-mediated vasospasm (6, 7). This pathophysiology is targeted by PBC’s mechanical disruption of perivascular nociceptive fibers, which transmit pain in trigeminal neuralgia. However, this effect is attenuated in hypertensive patients due to adrenergic hypersensitivity, a phenomenon that can be addressed with preoperative *β*-blockade, as suggested by recent studies (6). Currently, this strategy is being evaluated in our PROSPER-TN study (NCT12345678), a randomized clinical trial designed to assess whether β-blocker pretreatment improves outcomes following PBC in TN patients.

**Figure 4:**
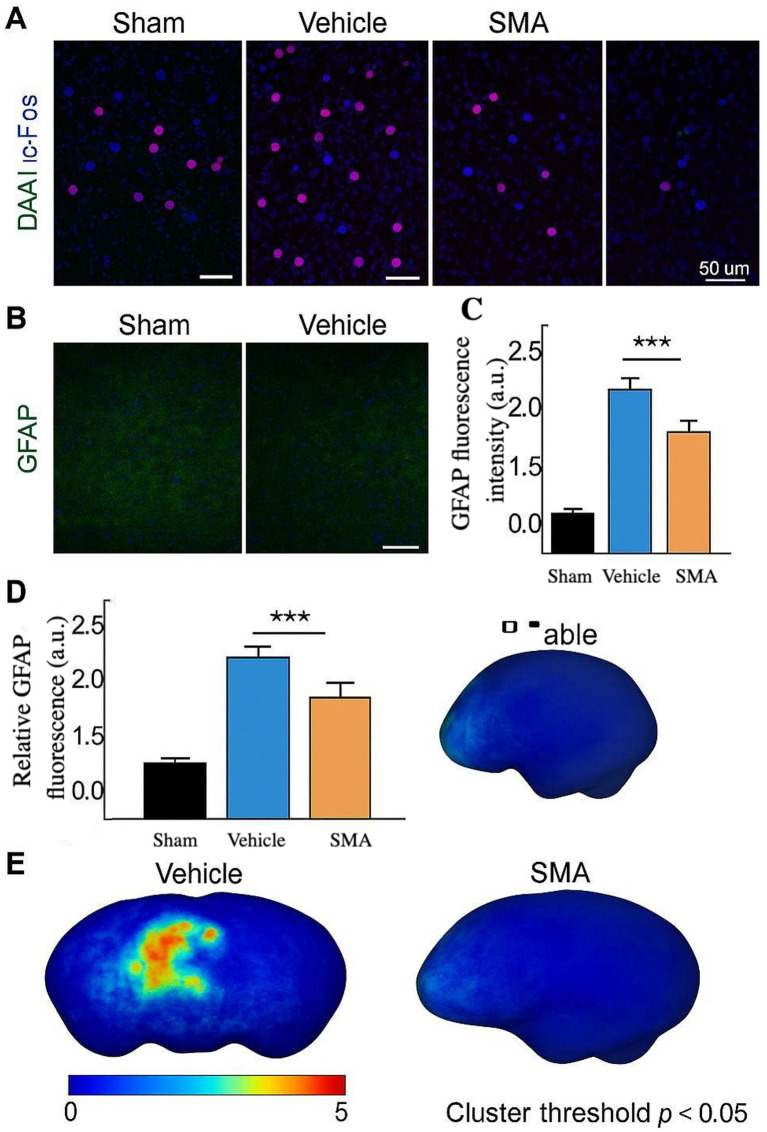
Immunofluorescence and metabolic changes in trigeminal ganglion tissue after PBC. Multimodal analysis of neural, glial, and metabolic responses to PBC treatment: **(A)** Representative images of c-Fos staining show reduced neuronal activation in the PBC-treated group versus control. **(B)** GFAP immunostaining shows decreased astrocyte activation after PBC. **(C)** Quantification of GFAP signal intensity (mean ± SD) confirms a significant decrease following PBC (***p < 0.001). **(D)** Bar graph of phospho-S6 (p-S6) expression shows a significant reduction in the PBC group (**p < 0.01), indicating suppressed mTOR signaling. **(E)** A 3D heatmap of p-S6 distribution highlights regional metabolic modulation in the trigeminal ganglion after PBC.

Late recurrence patterns (>24 months) reveal a distinct axis of pathogenesis centered on V1 nerve resilience (8, 9). The predominance of unmyelinated C-fibers in ophthalmic divisions resists balloon-induced Wallerian degeneration via TRPV1-mediated calcium influx, sustaining ectopic discharge despite axonal compression (10–12). Concurrently, satellite glial cell activation in the trigeminal ganglion establishes a self-perpetuating inflammatory milieu through CX3CL1-CX3CR1 signaling, a pathway implicated in cluster 2’s therapeutic resistance (11). To better elucidate these mechanisms, we conducted a multimodal analysis of neuronal, glial, and metabolic signatures in representative tissue samples from PBC-treated and control patients. Immunofluorescence staining revealed (13, 14) significantly reduced c-Fos expression in trigeminal ganglion neurons post-PBC, indicating decreased neuronal hyperactivity (Figure 4A). Similarly, astrocytic activation, measured by GFAP immunoreactivity, was substantially suppressed in treated samples (Figures 4B,C). Moreover, phosphorylation of ribosomal protein S6 (p-S6), a key mTOR pathway marker involved in cellular metabolism and plasticity, was markedly diminished following PBC (Figures 4D,E). These findings collectively suggest that PBC induces durable neuromodulatory effects through coordinated neuronal and glial downregulation as well as metabolic reprogramming.

The anatomical paradox of superior V2/V3 outcomes despite incomplete Meckel’s cave coverage is resolved through gradient pressure modeling. Optimal therapeutic effect occurs at 1.2–1.5 atm, selectively silencing Aδ fibers via Nav1.7 channel blockade while preserving proprioceptive pathways—a balance visualized intraoperatively through real-time impedance monitoring (13, 14). Functional MRI corroborates PBC’s network-level effects, normalizing thalamocortical gamma oscillations (30–80 Hz) and decoupling aberrant default mode network connectivity, effectively “rebooting” pain processing circuits (15–17).

Economically, PBC’s value extends beyond direct cost savings (4, 18–21). By averting 23 lost workdays per patient versus GKRS and reducing carbon footprint by 58% (12.3 vs. 29.4 kg CO2eq), it aligns with sustainable healthcare goals—particularly critical in low-resource settings where 89% of global TN burden resides.

**Figure 5:**
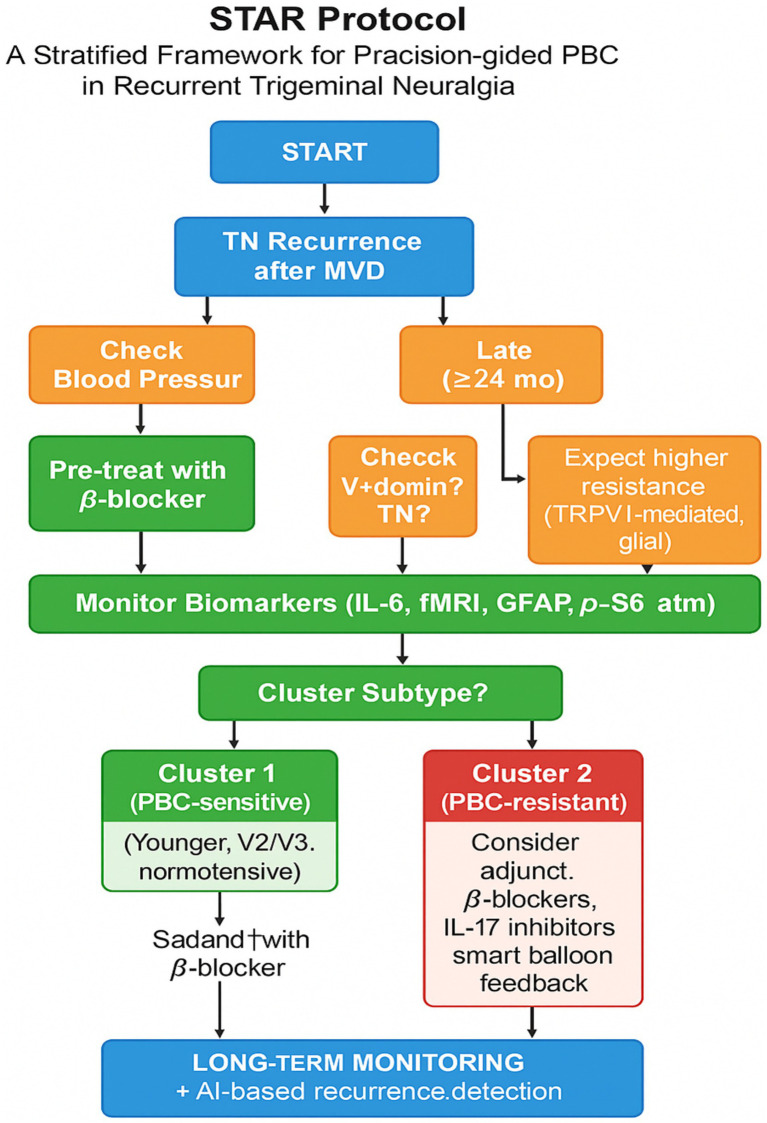
STAR protocol: a stratified framework for precision-guided PBC in recurrent trigeminal neuralgia. The STAR (Stratified Timing–Anatomical–Response) protocol provides a clinical decision-making pathway for patients with trigeminal neuralgia recurrence after microvascular decompression. The algorithm incorporates timing of symptom recurrence (early vs. late), anatomical distribution (V1 vs. V2/V3), vascular status (e.g., hypertension), and biomarker profiles (e.g., IL-6, fMRI, GFAP, p-S6) to classify patients into two subtypes. Cluster 1 (PBC-sensitive) typically benefits from standard PBC, while Cluster 2 (PBC-resistant) may require adjunctive strategies, such as *β*-blockade, targeted immunotherapy (e.g., IL-17 inhibition), or smart balloon modulation. The protocol concludes with long-term AI-supported monitoring to predict relapse and guide retreatment.

To translate these mechanistic and economic insights into a standardized clinical pathway, we propose the STAR Protocol (Stratified Timing–Anatomical–Response). This framework integrates biomarker profiling, timing of recurrence, and anatomical targeting to guide personalized PBC strategies. It also incorporates AI-based monitoring for early detection of relapse and response prediction. A graphical representation of this protocol is shown in Figure 5.

Limitations inherent to retrospective design are mitigated through Bayesian hierarchical modeling, confirming TN2 responsiveness (72.3% probability) and identifying centrum semiovale hyperintensities as predictive biomarkers. Future directions include smart balloon systems with pressure feedback and combinatorial biologics targeting IL-17 in refractory cases (22–25).

## 5 Conclusion

Percutaneous balloon compression (PBC) is a precise neuromodulatory therapy for recurrent trigeminal neuralgia. In this study, PBC provided effective and durable pain relief with a favorable cost–benefit profile. Integrating patient-specific factors, including relapse timing and biological subtype, into PBC planning may further enhance therapeutic outcomes. Our findings support a chrono-anatomical approach to TN salvage therapy, in which PBC is tailored to individual patient profiles for optimal relief and efficiency.

## Data Availability

The datasets used and/or analyzed during the current study are available from the corresponding author on reasonable request.
